# A Hybrid Feature Selection and Ensemble Approach to Identify Depressed Users in Online Social Media

**DOI:** 10.3389/fpsyg.2021.802821

**Published:** 2022-01-18

**Authors:** Jingfang Liu, Mengshi Shi

**Affiliations:** School of Management, Shanghai University, Shanghai, China

**Keywords:** depression, machine learning, ensemble learning, feature selection, social media

## Abstract

Depression has become one of the most common mental illnesses, and the widespread use of social media provides new ideas for detecting various mental illnesses. The purpose of this study is to use machine learning technology to detect users of depressive patients based on user-shared content and posting behaviors in social media. At present, the existing research mostly uses a single detection method, and the unbalanced class distribution often leads to a low recognition rate. In addition, a large number of irrelevant or redundant features in high-dimensional data sets interfere with the accuracy of recognition. To solve this problem, this paper proposes a hybrid feature selection and stacking ensemble strategy for depression user detection. First, recursive elimination method and extremely randomized trees method are used to calculate feature importance and mutual information value, calculate feature weight vector, and select the optimal feature subset according to the feature weight. Second, naive bayes, k-nearest neighbor, regularized logistic regression and support vector machine are used as base learners, and a simple logistic regression algorithm is used as a combination strategy to build a stacking model. Experimental results show that compared with other machine learning algorithms, the proposed hybrid method, which integrates feature selection and ensemble, has a higher accuracy of 90.27% in identifying online patients. We believe this study will help develop new methods to identify depressed people in social networks, providing guidance for future research.

## Introduction

Depression is a common mental disorder and is one of the main causes of disability and suicide worldwide. According to the World Health Organization, more than 300 million people worldwide suffer from depression (WHO, 2017), and the number has been increasing rapidly in recent years. Early treatment of depression can ameliorate the negative effects of the disease, but more than 70% of patients are reluctant to consult a doctor in the early stages of their illness (Shen et al., 2017). Stigma and discrimination cause them to be vigilant. Researchers have found that even if not every patient experiences such problems, stigma and discrimination are pervasive underlying problems (Whitley and Denise Campbell, 2014). Therefore, the identification of depression is a challenging task.

Relevant signals used to identify patients with depression have been detected in large real-time interactive social media data. For example, studies have found that depressed users tend to cluster with other potentially depressed users in their social networks (Vedula and Parthasarathy, 2017). In addition, the use of vocabulary can also be used as an indicator of depression. For example, in Twitter, verbs are more common in the text of users with depression, and first-person singular pronouns are also the most prominent feature of these patients (Leis et al., 2019). Therefore, many studies have targeted the analysis and characterization of depressed individuals and constructed predictive models to deal with a large amount of real-time interactive user data. In the early stages, De Choudhury et al. (2014) carried out pioneering work in this field to detect the risk of depression of users by measuring the relevant behavioral attributes of patients' social participation, emotion and language style. Although the statistical classification model they constructed did not achieve a high level of classification performance, their seminal work provided detailed working patterns for subsequent relevant studies. In recent years, deep learning has also attracted widespread attention. In Wang's study (Wang et al., 2020), three deep learning methods (BERT, Roberta, and XLNET) and a pretrained language expression model were adopted to predict the risk of depression. The data set included 13,993 Weibo posts with levels of depression annotated from 0 to 3. Among them, the Roberta method is more prominent in microblog depression detection.

At present, the research platforms for online depression detection are mainly focused on Twitter and Facebook (Islam et al., 2018; Alsagri and Ykhlef, 2020; Lin et al., 2020), and many researchers are trying to use different natural language processing methods to determine the characteristics of their contributions to build an accurate detection model. Due to the heterogeneity among social media, multiple features ranging from text and language to user-based and metadata can be extracted. From the perspective of language text, it usually involves multiple features, such as first-person pronouns, word frequency, LIWC dictionary, and sentiment analysis. The behavior patterns of users when participating in social media will involve posting time, number of posts, posting type (De Choudhury et al., 2013a, 2014; Hassan et al., 2017; Islam et al., 2018; Cacheda et al., 2019; Alsagri and Ykhlef, 2020), etc. Due to the diversity of data sources and the characteristics of text data, it is difficult to avoid the feature set featuring high dimensional sparsity. In order to reduce the complexity of computation, feature selection technology can be used as a preprocessing step of machine learning to eliminate irrelevant or redundant features, thus further improving the classification effect of the model. Dai et al. (2021) proposed a two-stage feature selection method to select the best feature subset. In the first stage, the maximum information coefficient (MIC) of multivariate filtering method is used to determine the significance ranking of features. In the second stage, the developed wrapper (SIER-SVM) was used to determine the optimal number of selected features, and finally the best performance was achieved in depression classification by using dozens of selected features. AlSagri and Ykhlef (2020) established a machine learning model based on random forest, and used a variety of technical methods to quantify the importance of features to calculate the importance of features. The experimental results show that the measurement method based on tree interpretation of feature importance can obtain higher classification accuracy.

Researchers often combine these features with different machine learning algorithms to improve their performance, such as Bayesian networks (Chomutare et al., 2015; Alsagri and Ykhlef, 2020), logistic regression (De Choudhury et al., 2014; Nguyen et al., 2014; Burdisso et al., 2019; Fatima et al., 2019; Thorstad and Wolff, 2019), support vector machines (De Choudhury et al., 2013a, 2014; Wongkoblap et al., 2018; Alsagri and Ykhlef, 2020), k-nearest neighbor algorithm (Islam et al., 2018; Burdisso et al., 2019), and neural networks (Benton et al., 2017; Dondena et al., 2017; Gkotsis et al., 2017; Kim et al., 2020; Lin et al., 2020). However, any single detection method has its own limitations. For example, Bayesian method has high constraint on attributes, which is often not valid in practical applications. Logistic regression is susceptible to interference from outliers, and its classification accuracy is not high. Support vector machines are not suitable for larger data sets and are more sensitive to missing data. The neural network model converges slowly, has a tendency to overfit, and has poor generalization ability. Generally, a combination of multiple base classifiers tends to have better generalization ability than a single classifier. In the study of Cacheda et al. (2019), according to different aspects of user writing (text spread, time gap, and time span) applied to detect early depression patients, the dual model method based on random forest proposed in this experiment improves the performance of the best detection model by over 10%. The model is formed by integrating multiple parallel-trained classifiers, each of which independently reaches the threshold and votes to determine the final result. In the study of Zhang et al. (2018), collaborative representation classifier (CRC) was used as the base classifier to solve the problems of insufficient samples and class imbalance, and Adaboost algorithm was used to carry out iterative training on it. Each weak classifier is assigned a different weight, and the process is repeated to achieve the best classification accuracy. However, the existing ensemble methods all contain the same type of base classifiers, which belong to isomorphic ensembles. If there is no difference or a high similarity between multiple basic classifiers in the ensemble method, there is no difference or similarity with a single classifier, and the performance may be worse.

There are still many unsolved challenges to online depression detection methods. First, compared with Twitter and Facebook, the detection of depressed users in Chinese communities is far from adequate. Second, the classification model used in many current studies is relatively limited. For different feature sets or data sets, the performance of the classifier is unstable, and there is a lack of research on the classification model. Therefore, this paper constructs a hybrid classification model suitable for identifying depressed users in social media to achieve better performance effects. Our contributions are as follows: (1) Taking Chinese social media (Weibo) as the research platform, combining Chinese language features and community functions, we summarized two types of characteristics of depressed users. (2) A hybrid feature selection method is designed to determine the optimal feature subset. Based on the stacking ensemble algorithm, a combined model was built to identify depressed users. In this model, support vector machine, naive bayes, k-nearest neighbour, and regularized logistic regression were used as the first-level basic learning model, and logistic regression was used as the second-level learners. (3) We compare the differences between multiple models and the stacking ensemble model on balanced and unbalanced data sets to demonstrate the advantages of the proposed approach. Our research can enrich the methods of identifying patients with depression in online social networks, especially to establish a set of functions selected based on Chinese social media, and integrate multiple single machine learning algorithms to establish a stable depression user identification model. In addition, this method can also be used to build related classification models in other fields.

## Materials and Methods

### Data Collection

Our dataset includes depressed users and other users, among which the depressed users are a sample collected under the microblogging supertopic “Depression,” which clusters a large number of active depressed patients under the community. In real life, depressed patients are often isolated or even rejected by others, whereas studies have shown that people with similar intrinsic distress choose to cluster together on social media. Therefore, our strategy for selecting depressed users is to search for user IDs posted on that topic, which can improve the efficiency of collecting samples of depressed users. For other user IDs, we chose to collect them under other active and living supertopics, such as “photography” under the photography section, “everyday” in the daily section, learning “study account” under that section, and “gourmet” in the food section. At this stage, all information from the user's personal interface is crawled once according to the collected user ID, including 315 depressive users and 562 other users.

The selection of samples will seriously affect the reliability of subsequent experimental results, so we reviewed the preliminary screening samples again. Under the “Depression” supertopic, there are many non-depressed users who only have mild depression or disguise their illness for other purposes. For depressed users, we need to clearly determine if they are real patients. The specific criteria are a confirmation of depression issued by users or the relevant pictures and text information about taking antidepressants. At the same time, to ensure the accuracy of the analysis, we eliminated community managers, marketing accounts, and accounts with original posting content below 60. Through the above strategies, we collected enough depressed user IDs and other user IDs and then obtained the personal interface information according to their user ID, including the number of users' fans, the number of followers, the number of posts, the post content, whether it is original content, post time, location information, publishing tools and other information fields. After rigorous sample screening, the final data sets of depressed users and other users were 130 and 320, respectively, and the numbers of microblog posts were 42,827 and 293,102, respectively.

### Ethics

The data used in the manuscript comes from the Chinese social platform-Sina Weibo, the data on this platform is publicly accessible, and users can choose whether to publish the information publicly. The raw data obtained does not include user data for setting privacy access. In addition, in order to protect the privacy of users, the data does not include personally identifiable information (such as user ID, user nickname).

### Feature Engineering

**Figure 1:**
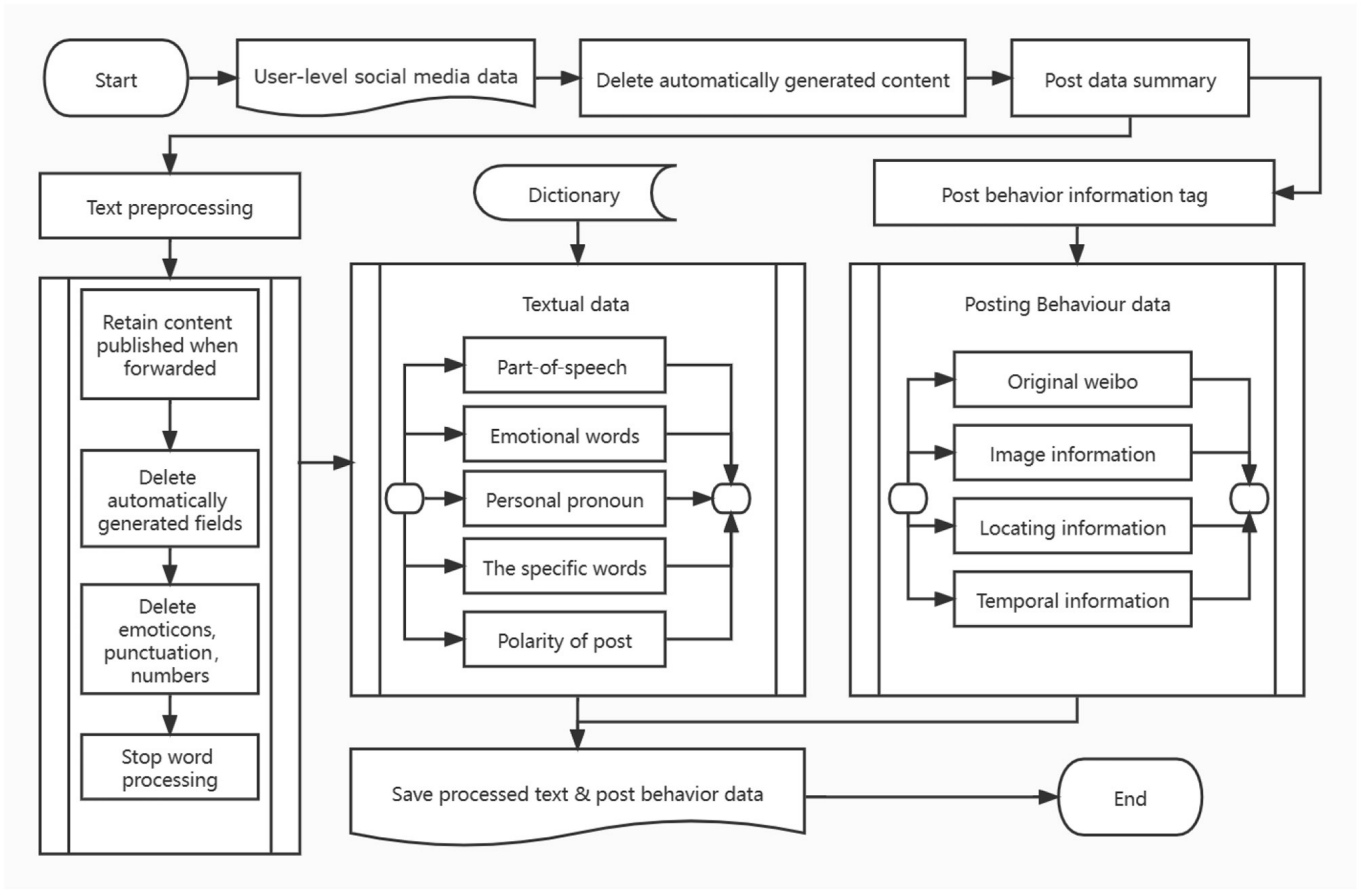
Data preprocessing and feature extraction.

Previous studies have proven that many functional features are very effective in distinguishing depressed patients from normal users, such as personal pronouns, negative words, and the patient's posting time. Combined with the existing research, microblog platform function and data field structure, feature engineering is divided into two aspects: text function and post behavior. Detailed information about the characteristics of the depression user prediction can be found in Table 1. In the part of speech features and personal pronouns section, we have added unique new features. At the same time, we extracted text features in the form of statistics to eliminate the influence caused by the large differences in the total number of posts among users. Specific data processing process is shown in Figure 1.

**Table 1:** Description of user characteristics.

**Group**	**Feature**	**Description**	**References**
Textual features	The part of speech	Percentage of 20 parts of speech in all posts by users	Islam et al., 2018
	Emotional words	The number of 7 types of emotional words classified according to the Chinese sentiment dictionary	Leis et al., 2019
	Personal pronoun	The frequency of singular/plural first-person pronouns, and the frequency of other pronouns	Vedula and Parthasarathy, 2017
	The specific words	Mainly include the number of negative words and interrogative pronouns	Leis et al., 2019
	Polarity	The emotional orientation of the post, 0 means negative, 1 means neutral, 2 means positive	Sadeque et al., 2018
Posting behavior	Posting habits	Proportion of original posts, posts with pictures and display positioning	Chen et al., 2020
	Posting time	The frequency of users post over a week and over a 24-h period	De Choudhury et al., 2013b

#### Text Features

As the part of speech is the basic grammatical attribute of words, part of speech tagging is also the foundation of natural language processing, so the accuracy of tagging sets determines the results of subsequent classification prediction. In order to improve the accuracy of word segmentation, we use Wikipedia based anti depression (http://en.wikipedia.org/wiki/List_of_antidepressants) build a dictionary list of drugs for depression to improve the accuracy of word segmentation. At the same time, some common network terms and abbreviations have also been added to the dictionary. In addition, most studies use the part of speech of LIWC text to mark, while LIWC is mainly oriented to English text information, and there are great differences in the way of expression and language style between Chinese and English. We use the Chinese word segmentation tool Jieba (https://github.com/fxsjy/jieba) to mark the part of speech of each word, 20 classes are selected Chinese parts of speech, including some unique part of speech in Chinese language environment, such as onomatopoetic words, idioms, interjection, etc. Abnormal emotional preference is one of the important symptoms of patients with depression. The frequency of emotional words of different categories can be used as important information to distinguish different users. According to the Emotion Dictionary of Dalian University of Technology (Xu et al., 2008), seven kinds of fine-grained emotions that appeared in user posts were counted, namely, happy, like, anger, sad, fear, surprise and disgust. Previous studies have shown that personal pronouns reflect the psychological distance between depressed users and others (Vedula and Parthasarathy, 2017). Here, we added 'others' as a personal pronoun feature, which we believe to be valid based on previous research. Specific words mainly include negative words and the frequency of interrogative words. In previous studies, the number of negative emotional posts was usually calculated to identify the depression tendency of users (Sadeque et al., 2018), while the proportion of posts with different polarities was used to distinguish them in this study. Because the catharsis of negative emotions is not completely the same as depression, only when the negative posts reach a certain proportion can the painful mental state of the users be reflected. Here, we chose to use the text sentiment analysis API of the Baidu Intelligent Cloud Platform to mark the polarity of all original posts.

#### Characteristics of Posting Behavior

A study on Facebook found that non-original posts in users' status updates are strongly associated with depression (Chen et al., 2020). We separately counted the percentage of each user's original posts in the total number of posts and used it to reflect the user's posting habits. By observing the post behavior of different users on the platform, it was found that patients with depression prefer to express their feelings and mental status through words and less visual information (e.g., pictures). In addition, based on the functional characteristics of the microblog platform, we increased the posting frequency of users to display location information. The posting time is considered to be a reflection of the user's daily schedule, so we will compare the posting rates of users at different times, which are every 6 h in a 24-h day and time periods with “week” intervals. Early in the morning is the peak of depression, so late at night, depressed users post more frequently than other users. In addition, depressed users rely heavily on social media to vent their pain, and whether it is a workday or not may significantly affect the frequency of the patients' posts, so we believe this feature will be effective in providing useful information.

### Methods

**Figure 2:**
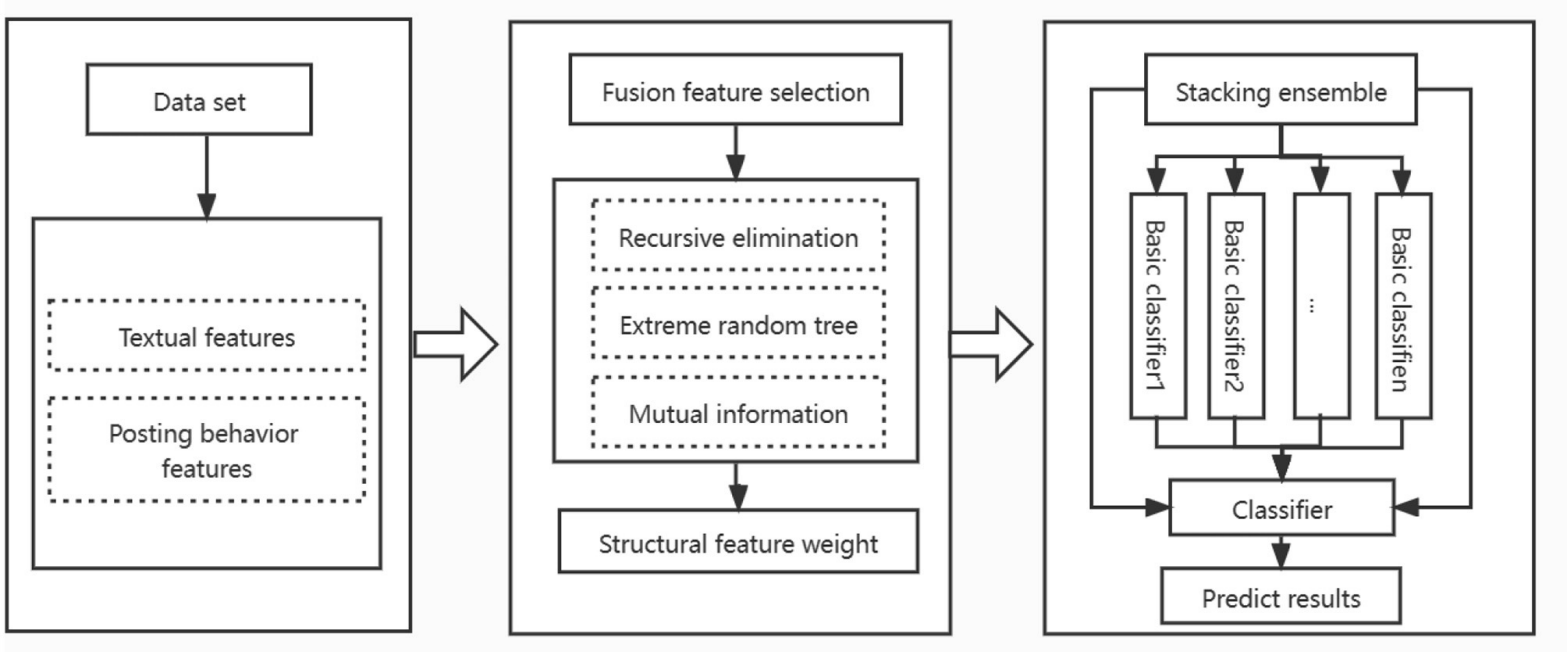
Depression user identification framework.

In order to improve the recognition ability of depressed users in online social media, we proposed a recognition framework of hybrid feature selection and ensemble learning strategies. In the feature selection stage, a feature selection method combining multiple modes is designed. This method uses recursive elimination method and extremely randomized trees method to get feature importance, combines mutual information method to get the importance score of each feature, and eliminates the feature with lower score after weight fusion to get the best feature subset. In the classification stage, a subset of the identified features is input into the stacking ensemble model to classify. Accuracy, f1-measure, precision and recall are used as evaluation criteria in the experimental phase, and the robustness of the model was measured by a 10-fold cross validation. Figure 2 shows the proposed method identification framework, which consists of the following three phases:

Dataset processing stage: First, the collected data is preprocessed to extract functional features from two perspectives of user's text and posting behavior information, and then convert them into data formats suitable for analysis.

Feature selection stage: In order to avoid the impact of high-dimensional sparsity of original data on the classification effect of the model, this paper adopts a hybrid feature selection method, which combines the advantages of wrapper, filter and embedded methods to eliminate the feature vectors with low relevance or redundancy in the identification of depressed users.

User classification stage: In order to improve the identification accuracy of depressed users, we use naive bayes, k-nearest neighbor, regularized logistic regression and support vector machine as basic learners, and use simple logistic regression algorithm as combination strategy to establish an ensemble model.

#### Mixed Feature Selection

Feature selection methods (Guyon et al., 2006) can apply search techniques to obtain new feature subsets from a given data set and evaluate the scores of subsets of different feature subsets. Feature selection can remove irrelevant and redundant features, eliminating the disaster caused by the dimension curse. Reducing the functional space not only helps to build more accurate prediction models, but also reduces training time and improves model generalization ability. In practice, we cannot determine the only optimal feature subset, and different feature subsets may produce the best classification effect for different machine learning algorithms. Therefore, the feature selection technology has been the focus of researchers' attention, currently mainly including three types of methods: wrapper, filter and embedded (Guyon and Elisseeff, 2003). The wrapper uses a machine learning algorithm to select a subset of features and iterate over all remaining features each time. The filter method uses statistical techniques to evaluate the relationship between features and target values, independent of machine learning algorithms, and classification performance can be used as evaluation criteria for selecting feature subsets for wrapper method. Compared with the wrapper method, the embedded method enables feature selection and algorithm training to be carried out at the same time, and the computational cost is lower, but it is not easy to obtain higher performance. The recursive feature elimination method (RFE) based on random forest classifier is selected for wrapper method, which has the ability to select predictive variables with higher accuracy. The mutual information method is one of the most important methods in the filter method and can be used as a measure of the interdependence of variables. In the field of machine learning, the explanatory nature of tree far exceeds that of complex models such as neural network, so we choose extremely randomized trees to calculate the importance score of characteristics.

A single feature selection method may ignore other potential information contained in the original feature set, thus affecting the classification effect (Bolón-Canedo and Alonso-Betanzos, 2019). We chose to combine the advantages of multiple feature selection methods and apply each feature selection method to all feature sets. Among them, recursive elimination based on random forests and embedding based on extra-trees yielded weight vectors of importance for *n* features, respectively, $W_{r}=|\begin{array}{c} R_{1},\cdots \;,R_{n} \end{array}|$ and $W_{e}=|\begin{array}{c} E_{1},\cdots \;,E_{n} \end{array}|$. The mutual information method can calculate the mutual information values for each feature. In order to further obtain the weight vectors of each feature, we calculated the proportion of each feature's mutual information value in the sum of all the features, and obtained $W_{m}=|\begin{array}{c} M_{1},\cdots \;,M_{n} \end{array}|$. Using a fusion strategy *W* = *W*_*r*_ + *W*_*e*_ + *W*_*m*_, the weights obtained in three different ways were ranked and the 20% features with lower weights were removed. By using the fused feature selection method, the feature vectors most relevant to identifying depressive patients are selected to avoid the loss of effective information and reduce the feature dimension.

#### Ensemble Learning

The concept of ensemble generalization was originally proposed by Wolpert (1992). Its core idea is to build multiple basic learning models, and use the output information of the basic prediction models to combine into a more powerful prediction model for final decision-making. The stack ensemble is a framework of layered combinatorial models. To be more precise, there are usually two stages in stack integration. The first stage consists of several basic models, which are trained separately on the training set, thus establishing the first level of prediction in the stack system. The predicted values for this phase are then collected as a data set for the next phase. In this new data set, the output values of each model in the first stage constitute the new feature items. In the second stage, the new data set is used as the feature input to the secondary model for training, while the prediction results of the test set in the first stage are used as the test set for prediction, and the final learning results are output. In fact, stack ensemble can group the heterogeneity of multiple basic models into one and combine the prediction results of basic models to reduce the generalization error (Pernía-Espinoza et al., 2018). Therefore, the combination of basic learning models can be effectively deployed to reduce the bias and improve the prediction accuracy.

Multiple basic classifiers can improve the heterogeneity of the stacking ensemble classification method, and the lower the correlation between them, the generalization ability of the model can be effectively improved. We use different types of single classification models for experiments, including k-nearest neighbour (KNN), lasso regression (LG1) and ridge regression (LG2), decision tree (DT), naive nayes (NB), multinomial naive bayes, gaussian bayes, and support vector machines (SVM). The final results show that KNN, NB, LG1, LG2, and SVM algorithm have better results. Although they have certain deficiencies under certain conditions, they have always held their place in the problem of text classification because they also exhibit their own advantages in different aspects based on different principles. Considering the size of our data and the properties of our data set (containing a large amount of text information) together, our selected research methods are more in accordance with the characteristics of this research question. In the second stage, we choose a simple binary logistic regression model to combine different basic models. Although other classification models can be used, choosing binary logistic regression can effectively avoid overfitting and help improve the performance of the model (Whalen and Pandey, 2013). In the binary logistic regression, the probability of the user being identified as a patient is taken as the prediction index by the five models combined in the first layer, and the final prediction result is determined by the model.

To provide a clearer interpretation of the learning framework of the stacking ensemble approach, we design an illustrative case. The case includes 11 samples, as shown in Table 2. Assume that samples $\left[\begin{array}{c} X1,\cdots \;,X10 \end{array}\right]$ is the train set and X11 is the test set. In the first layer of prediction, the SVM does samples $\left[\begin{array}{c} X3,\cdots \;,X10 \end{array}\right]$ and the original labels $\left[\begin{array}{c} Y3,\cdots \;,Y10 \end{array}\right]$ as training set input model for training, and uses the SVM model obtain to predict the samples $\left[\begin{array}{cc} X1, & X2 \end{array}\right]$, resulting in $\left[\begin{array}{cc} S1, & S2 \end{array}\right]$. By training five times in turn, we obtain the prediction results $\left[\begin{array}{c} S1,\cdots \;,S10 \end{array}\right]$ of samples $\left[\begin{array}{c} X1,\cdots \;,X10 \end{array}\right]$ under SVM. Meanwhile, five SVM models are used to predict the test set sample X11, and the label S11 is obtained. Similar to the process of SVM, KNN, NB, LG1 and LG2 also get the prediction results of each sample under their respective models. In the second layer of processing, the prediction results $\left[\begin{array}{ccc} S1 & \cdots & R1\\ \vdots & \ddots & \vdots \\ S10 & \cdots & R10 \end{array}\right]$ of the first layer are entered as features and the real labels $\left[\begin{array}{c} Y1,\cdots \;,Y10 \end{array}\right]$ together into the meta classifier logistic regression training, and then the prediction result $\left[\begin{array}{cc} S11, & \begin{array}{ccc} K11, & N11, & \begin{array}{cc} LA11, & R11 \end{array} \end{array} \end{array}\right]$ of the test set sample Y11 of the first stage are entered into the trained logistic regression model to get the final prediction result L11.

**Table 2:** Example of stack integration.

**Samples**	**The first level**					**The second level**	**Label**
	**SVM**	**KNN**	**NB**	**LG1**	**LG2**	**LG**	
X1	S1	K1	N1	LA1	R1		Y1
X2	S2	K2	N2	LA2	R2		Y2
X3	S3	K3	N3	LA3	R3		Y3
X4	S4	K4	N4	LA4	R4		Y4
X5	S5	K5	N5	LA5	R5		Y5
X6	S6	K6	N6	LA6	R6		Y6
X7	S7	K7	N7	LA7	R7		Y7
X8	S8	K8	N8	LA8	R8		Y8
X9	S9	K9	N9	LA9	R9		Y9
X10	S10	K10	N10	LA10	R10		Y10
X11	S11	K11	N11	LA11	R11	L11	Y11

## Results

### Relative Importance of Analysis Features

As mentioned above, we design a feature selection method that combines recursive elimination based on random forest, extremely randomized trees and mutual information. Different methods have different emphases in feature selection, so the loss of important information can be avoided from multiple perspectives. The recursive elimination method and the extra-trees method get the importance score of features, while the mutual information method gets the mutual information value of features. To make the feature mutual information values comparable, we computed feature weight ratios to fuse the results of the three methods. By excluding the 20% of features with low scores, we obtained the best feature subset, including a total of 42 features.

**Figure 3:**
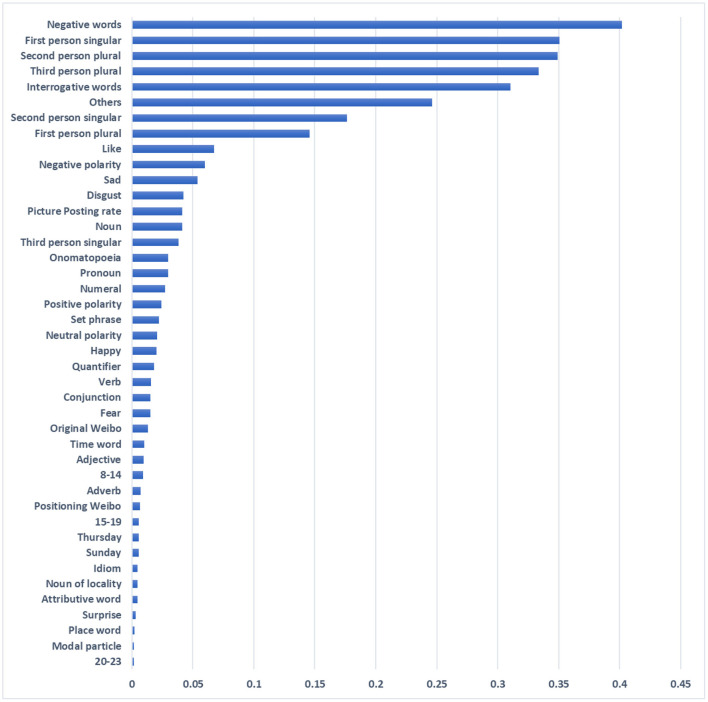
Relative importance of features.

Figure 3 shows the importance of features as determined by our feature selection strategy. It can be observed from the figure that features with significant influence on depression users' recognition include negative words, first-person singular, second-person plural, and interrogative words. Existing studies have shown that depressed users use more first-person pronouns (De Choudhury et al., 2014), reflecting their high degree of self-concern and self-suggestion. Another interesting finding was that the word 'others' was also good at discriminating, suggesting that patients may subconsciously maintain psychological distance from those around them. In addition, the Chinese parts of speech (such as onomatopoeic words and set phrase etc.) involved for the first time in this study also have outstanding contributions, which can make further detailed analysis in future research. There was also a subliminal change in users' posting habits before and after their illness, such that there was some effect on their frequency of posting pictures, original microblogs, and positioning microblogs. These behaviors typically expose more private information, and users reduce the disclosure of such information due to their own stigma.

### Performance of the Baseline Experiment and the Ensemble Experiment

This section mainly compares and analyses the results of baseline experiments and ensemble experiments of multiple models. The baseline experiments mainly included five traditional machine learning algorithms, support vector machine (SVM) (Vapnik, 1998), naive bayes (NB) (Hand and Yu, 2001), k-nearest neighbor algorithm (KNN) (Cover and Hart, 1967), lasso regression (LG1) and ridge regression (LG2) (Theil, 1969), which have been used in other studies to identify mental illness. In the ensemble experiment, we superimposed a variety of baseline experiment methods for measurements. The superparameter of the model is the external configuration of the model, which cannot be estimated from the data itself. However, the choice of the parameter is crucial since it controls the overall behavior of the model. Considering that each algorithm involves many different superparameters, we used the method of network searching to determine the best combination of parameters. In the five baseline experiments, 75% of the data were used for training and 25% for testing, and the optimal parameter combination of each model was determined by the method of 10-fold cross-validation. At the same time, the optimal parameters were used in the first layer learner of the stack ensemble, and the second layer learner also used the network search method to select the optimal parameters. Appropriate parameters can improve the accuracy of the model, but the key problem is that there is no constant superparameter in any data, so we need several grid searches to determine the best value among the different experimental data. Our experiment includes three classification models: (1) a model with only text data, (2) a model that includes posting behavior information, and (3) a model that includes text characteristics and posting behavior data. The classification prediction problem includes a variety of performance metrics, and different classification tasks are suitable for choosing different indicators to measure. We chose accuracy, f1-measure, precision and recall as the metrics for performance evaluation.

We constructed different model sets, and each method was trained for different feature sets. Table 3 details the results of the NB, KNN, SVM, LG1, LG2, and ensemble experiments on the three models. When all of the features were added (model 3), the optimal results of the baseline experiment is KNN (0.8761), but the ensemble method is better than this value (0.9027), which is 2.66% higher than the baseline optimal results. This shows that the ensemble method is more effective than the single method. Although the results of the five baseline experiments achieved a reasonable performance, there is still potential for improvement.

**Table 3:** Performance measurements of each method on different models.

**Model**	**Indicators**	**SVM**	**NB**	**KNN**	**LG1**	**LG2**	**Stacking**
Model1 (textual)	Precision	0.7339	0.8068	0.8750	0.8444	0.8462	0.8750
	Recall	0.9999	0.8875	0.9625	0.95	0.9625	0.9625
	F1-measure	0.8465	0.8452	0.9166	0.8941	0.9006	0.9166
	Accuracy	0.7434	0.7699	0.8761	0.8407	0.8496	0.8761
Model2 (posting behavior)	Precision	0.7080	0.8434	0.8256	0.8202	0.8242	0.8256
	Recall	1.0000	0.8750	0.8875	0.9125	0.9375	0.8875
	F1-measure	0.8290	0.8589	0.8554	0.8639	0.8772	0.8554
	Accuracy	0.7080	0.7965	0.7876	0.7965	0.8141	0.7876
Model3 (textual + posting behavior)	Precision	0.8478	0.8142	0.8750	0.8539	0.8556	0.8791
	Recall	0.9750	0.9000	0.9625	0.9500	0.9625	1.0000
	F1-measure	0.9070	0.8727	0.9166	0.8994	0.9059	0.9357
	Accuracy	0.8584	0.8142	0.8761	0.8496	0.8584	0.9027

It can be observed in Table 3 that the performance of the single and ensemble methods on only text characteristics is mostly better than the models with only posting behavior characteristics; that is, the accuracy of model (1) is generally better than that of model (2), except for the naive bayes algorithm. At the same time, it can be noted that model (3) to model (1) has a better predictive effect, and the table also reflects that language characteristic information has a more significant contribution in predicting depressed users than the posting behavior models.

**Figure 4:**
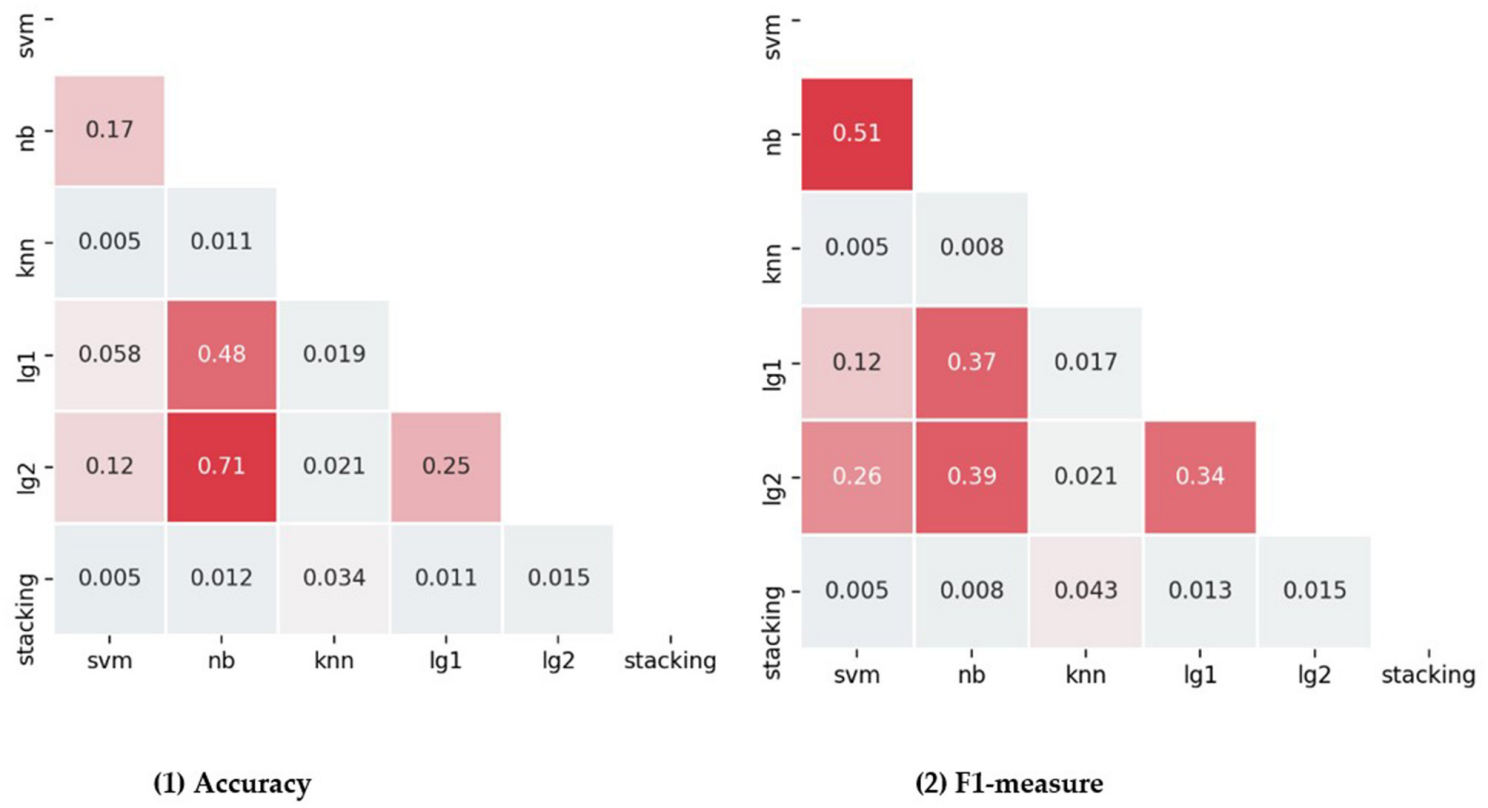
Pairwise compares each classifier and ensemble method in terms of accuracy and f1-measure.

In addition, statistical tests were used to compare the addition of all features. The results of Friedman test for all individual classifiers and integration methods showed significant differences in accuracy [$\chi_{\left(6\right)}^{2}$ = 28.475, *p* < 0.001], F1-measure [$\chi_{\left(6\right)}^{2}$ = 27.781, *P* < 0.001], Precision [$\chi_{\left(6\right)}^{2}$ = 31.040, *P* < 0.001], Recall [$\chi_{\left(6\right)}^{2}$ = 30.472, *P* < 0.001]. Wilcoxon test was used to compare the accuracy and f1-measure values of each method. The comparison results are shown in the Figure 4. The greater the *P*-value in the figure, the greater the saturation of the red color. The pairwise comparison of accuracy and f1-measure value shows that the stacking ensemble is superior to other classifiers, and the difference is significant.

In general, several key conclusions can be drawn from the analysis. First, the fusion feature selection and stacking ensemble model we designed further improves the prediction of every single algorithm. In some cases, the stack ensemble algorithm can complement the advantages of multiple classifiers; that is, the disadvantages of a single classifier on some features will be compensated by other classifiers. Second, both the text characteristics and the posting behavior characteristics have a positive impact on the performance of the system. However, language characteristic information has a stronger predictive ability than posting behavior information, so text characteristics of depression may reflect more information than other features.

### The Impact of Class Imbalance on Prediction Accuracy

Classifiers face a challenging problem in the field of data mining; that is, the distribution of classes in practical problems is often unbalanced. This kind of situation is ubiquitous, such as fault detection (Velandia-Cardenas et al., 2021), image processing (Bahrami and Sajedi, 2019), text classification (Sarakit et al., 2015), and medical diagnosis (Singh et al., 2021), so it has also attracted extensive attention from researchers in practical applications. Usually, the number of samples in one class in the dataset is far less than the number of other samples. In previous studies, it has been proven that an unbalanced data distribution has a significant impact on the accuracy of model prediction (Daskalaki et al., 2006). To observe the impact of the unbalanced distribution of observation categories on the six classifiers identified in this paper, we need to ensure that the data partition is representative. In the previous section, stratified sampling was used to divide the training set and the test set to keep the data structure unchanged. In other words, the ratio of the test set and the training set for depressed and non-depressed users is consistent with the original data set and is unbalanced. In this section, we built balanced class distribution data from the original unbalanced dataset and compared the changes in the prediction accuracy of each classifier.

The synthetic minority oversampling technique (SMOTE) is one of the representative methods in oversampling technology (Chawla et al., 2002). Its basic idea is to create new minority sample instances by interpolating between minority samples. For each sample in the minority class, the nearest K to the sample set of the minority class is calculated. A sample is randomly selected according to the sampling rate, and a new sample is synthesized by random interpolation between the two samples. This method can extend the decision boundary of a few class samples to a majority class sample space, thus avoiding overfitting to a certain extent. Based on SMOTE, we created a balanced set of data sets and trained six classifiers in the same way. Table 4 shows the prediction accuracy of different classification models in unbalanced and balanced data sets. The results show that the accuracy of LG1 model has the most significant change from unbalanced data to balanced data, increasing by 8.17%, while the proposed method has a smaller improvement, increasing by 5.36%. After eliminating the class imbalance, the prediction accuracy of both the baseline model and the ensemble model improved, and the accuracy of the stacking emsemble experiment reached 0.9563. Compared with the best LG1 model in baseline experiment, the accuracy is improved by 2.5%. Therefore, the method proposed in this paper can make the prediction performance of the model get better results.

**Table 4:** Prediction accuracy of classifiers before and after eliminating class imbalance.

	**Accuracy of unbalanced data**	**Accuracy of balanced data**	**Improve (%)**
SVM	0.8584	0.8688	1.04%
NB	0.8142	0.8875	7.33%
KNN	0.8761	0.8938	1.77%
LG1	0.8496	0.9313	8.17%
LG2	0.8584	0.8750	1.66%
Stacking	0.9027	0.9563	5.36%

### Compare With Other Methods

**Figure 5:**
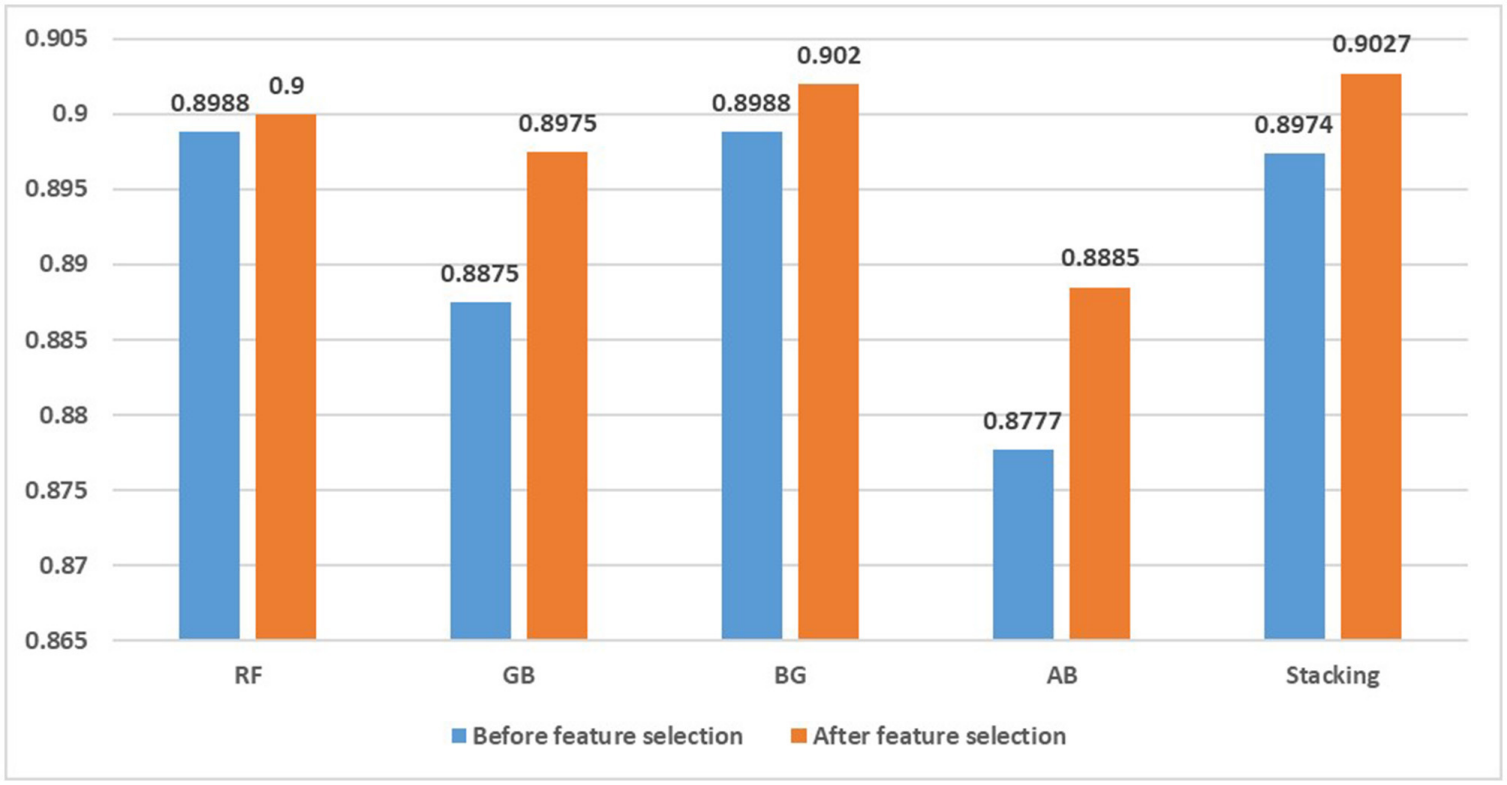
Accuracy of integration method before and after feature selection.

Finally, to further validate the validity of the proposed models, we tested the results of the proposed methods compared with other ensemble experimental methods, including random forest (RF), gradient boosting (GB), bagging (BG) and adaboost (AB), which performed well in previous depression recognition and text analysis studies (Zhang et al., 2018; Cacheda et al., 2019; Budhi et al., 2021). The default parameters are chosen for each method, and the results are checked using a 10-fold cross-validation method. Figure 5 shows the accuracy values of random forest, gradient boosting, bagging, adaboost and the stacking ensemble model we constructed before and after applying mixed feature selection. The results show that the stacking method gives better results than other ensemble models. Bagging has an accuracy of 0.9020 as a classifier in identifying depression, while our model achieves the best performance (0.9027). At the same time, we find that the feature fusion method of text design is applied to various integration models and the classification effect is improved to a certain extent. So this paper fused feature selection and integration method has a certain competitiveness in identifying depression users and can improve the performance of classification model.

## Discussion

The diagnosis of depression is quite complicated and time-consuming, while the detection of psychological diseases through the network community has provided researchers with new ideas. Currently, various single classifiers have been deployed to identify depressed patients in online social media, but most results were unstable or unsatisfactory due to the phenomenon that the datasets presented high-dimensional sparse features and class imbalance. In this study, we propose a novel online social media depression user detection framework based on feature selection and ensemble learning. First, we fused multiple feature extraction methods with the goal of identifying the features most closely related to depressed users. This method covers the advantages of wrapper, filter, and embedded three-class methods, compensating for the lack of optimal feature subsets identified in different ways. The feature weight vector was determined by hybrid recursive elimination method, based extra-trees as well as mutual information method to take full advantage of each method and avoid the information loss of effective features. At this stage, we identified the 42 most relevant features of depression, including some new features that we have added to incorporate the Chinese language style and social platform features, such as onomatopoeia, “others” and location information posting frequency. In the future, we can further analyze the language styles of depressive patients in different language environments. For example, onomatopoeias are often used in Chinese to describe specific sounds and things, reflecting a relaxed mood, so depressed users rarely use onomatopoeias.

Second, in order to better identify depressive patients in online social media, we build an ensemble model by overlaying five traditional classifiers, SVM, NB, KNN, LG1, and LG2 to solve this problem at the algorithmic level, thus improving the accuracy of the model. These basic classifiers have been widely used in depression detection with high accuracy. At the same time, heterogeneity helps to improve the accuracy of the ensemble model. There are some differences among the five baseline models, and different learning strategies are used to learn feature space from different angles to achieve the model complementarity. In the original class unbalanced dataset, the stacking ensemble model has achieved significant performance improvement compared with the other five baseline models, with an accuracy of 0.9027. Because in some cases the stacking ensemble algorithm can make up for the weaknesses of several classifiers, that is, the shortcomings of a single classifier on some features will be remedied by others. In addition, the experimental results showed that compared with the model with only posting behavior information, the accuracy of text information in identifying depressed patients increased by 8.85%. Therefore, text characteristics of patients play a more important role than posting behavior information in identifying depressed users. The results suggest that user-generated content has great potential for detecting depression and tracking their mental health.

Finally, we analyze the effect of category imbalance on the prediction accuracy of classification model. The results show that the ensemble model still performs better than the single model after eliminating the class imbalance, achieving an accuracy of 0.9563 in the balanced data set. To further verify that the combination of feature selection and ensemble methods can improve the detection level of depressed users, we compared the proposed method with other ensemble algorithms, including random forest, gradient boosting, bagging and adaboost. The results show that before feature selection, the performance of random forest and bagging are better than that of our model, achieving an accuracy of 0.8988. After applying the hybrid feature selection method designed in this paper, our results are better than other ensemble classification algorithms, and the performance of different ensemble classification algorithms is improved to different degrees. Therefore, we conclude that the detection framework that combines feature selection and stacking ensemble method is more suitable for the identification of depressed users and has a strong competitive advantage.

This paper draws some valuable conclusions, but there are still some limitations. First, due to the younger age of depressive users, the majority of users participating in the microblog depression community are adolescents, and the sample studied may not represent the general population. At the same time, the users of the control group may also suffer from other mental diseases, so there are some limitations in the promotion of the current findings. Second, our study can provide a preliminary judgment to some users who have difficulty in determining whether they are diseased or not, and it is difficult to avoid misidentification cases. We cannot fully understand other symptoms and reality of patients through social media. In future studies, we hope to further improve recognition accuracy by combining social media data with clinical data. Finally, due to the limitation of the sample data of depressed users, we are unable to conduct large-scale deep learning algorithm research. Therefore, in subsequent studies, researchers can enrich the specialization and richness of the data set. In terms of data, future research objectives may provide targeted research for different user types, such as gender, age and family composition. In addition to user differences, different types of depression may also have differences or common factors, such as seasonal emotional disorders, bipolar emotional disorders and postpartum depression. At the method level, future research can integrate more unique learning methods to explore whether different variables or parameters can produce higher predictive performance than previous advanced ensemble methods.

## Data Availability Statement

The raw data supporting the conclusions of this article will be made available by the authors, without undue reservation.

## Ethics Statement

Ethical review and approval was not required for the study on human participants in accordance with the local legislation and institutional requirements. Written informed consent from the participants' legal guardian/next of kin was not required to participate in this study in accordance with the national legislation and the institutional requirements.

## Author Contributions

JL: methodology. MS: validation, data curation, writing—original draft preparation, and visualization. JL and MS: writing—review and editing. All authors have read and agreed to the published version of the manuscript.

## Funding

This research was funded by the Natural Science Foundation of Shanghai, Grant Number 19ZR1419400.

## Conflict of Interest

The authors declare that the research was conducted in the absence of any commercial or financial relationships that could be construed as a potential conflict of interest.

## Publisher's Note

All claims expressed in this article are solely those of the authors and do not necessarily represent those of their affiliated organizations, or those of the publisher, the editors and the reviewers. Any product that may be evaluated in this article, or claim that may be made by its manufacturer, is not guaranteed or endorsed by the publisher.
